# What is Weight Loss after Bariatric Surgery Expressed in Percentage Total Weight Loss (%TWL)? An Updated Systematic Review

**DOI:** 10.1007/s11695-026-08766-3

**Published:** 2026-06-12

**Authors:** Rutger Franken, Felix Hers, Redouan El Ajraoui, Kelly Tieken, Ralph de Vries, Dennis Souverein, Max Nieuwdorp, Victor Gerdes, Arnold van de Laar

**Affiliations:** 1 Department of Surgery, Spaarne Gasthuis, Haarlem, Netherlands; 2 Medical Library, Amsterdam UMC Location VUmc, Amsterdam, Netherlands; 3 Regional Public Health Laboratory Kennemerland, Streeklab Haarlem, Haarlem, Netherlands; 4 Department of Vascular Medicine, Amsterdam University Medical Centers, Amsterdam, Netherlands; 5 Department of Internal Medicine, Spaarne Gasthuis, Haarlem, Netherlands

**Keywords:** Bariatric surgery, One anastomosis gastric bypass, Roux-en-Y gastric bypass, Sleeve gastrectomy, Percentage total weight loss

## Abstract

Percentage total weight loss (%TWL) is a robust and widely used outcome measure for assessing weight loss after metabolic and bariatric surgery (MBS). This study is an update of the 2021 systematic review on weight loss after MBS expressed in %TWL, now including results of one anastomosis gastric bypass (OAGB), Roux-en-Y gastric bypass (RYGB), and sleeve gastrectomy (SG). A total of 4,541 records were screened, and 49 studies were included, comprising both cohort and registry-based data. Descriptive pooled estimates suggested that OAGB was associated with the highest nadir %TWL within the first two years, whereas nadir %TWL appeared comparable between RYGB and SG. During longer-term follow-up, OAGB appeared to maintain a higher pooled mean %TWL than RYGB and SG. These findings should be interpreted cautiously given the heterogeneity of included studies and the limited availability of long-term data. They should be balanced against long-term complications and need for revisional surgery, which were beyond the scope of the present review. Standardized reporting and high-quality studies with extended follow-up using %TWL as the primary outcome are needed to improve comparability and interpretation of long-term weight loss after bariatric surgery.

## Key points

- Early (≤ 2-year) %TWL appeared highest after OAGB in descriptive comparison with RYGB and SG.
- Available long-term data suggest sustained %TWL after OAGB compared with RYGB and SG, although direct comparisons remain limited by heterogeneity and incomplete follow-up.
- Substantial heterogeneity in patient characteristics, operative techniques, limb lengths, and outcome reporting limits robust comparison between bariatric procedures.
- Standardized long-term reporting of weight loss, complications, and revisional surgery is needed to improve comparability across bariatric procedures.

## Introduction

Weight loss remains the most commonly reported outcome after metabolic and bariatric surgery (MBS). Among available metrics, percentage total weight loss (%TWL) is increasingly considered one of the most robust and clinically interpretable measures and has been endorsed by the Dutch Society for Metabolic and Bariatric Surgery (DSMBS) [1]. In addition, %TWL is widely used in contemporary pharmacological obesity trials, facilitating comparison between surgical and non-surgical treatments. In 2021, Van Rijswijk et al. published the only systematic review summarizing postoperative weight loss expressed in %TWL after Roux-en-Y gastric bypass (RYGB) and sleeve gastrectomy (SG) [2]. Since then, one anastomosis gastric bypass (OAGB) has been increasingly adopted, and recent studies have reported substantial weight loss following this procedure [3–8]. In parallel, pharmacological obesity treatments, including GLP-1 receptor agonist–based therapies, have increasingly reported weight loss outcomes in %TWL. Therefore, an updated review is warranted. The aim of this study was to update the available evidence on postoperative weight loss after bariatric surgery expressed specifically in %TWL. This review focuses on weight-loss outcomes and was not designed to compare procedures across all anthropometric or metabolic outcome measures. Accordingly, pooled values should be interpreted as descriptive summaries of the available literature rather than formal comparative effect estimates.

## Materials and Methods

This review is reported according to the Preferred Reporting Items for Systematic Reviews and Meta-Analyses (PRISMA) (www.prisma-statement.org) [9]. The review was registered at PROSPERO on august 24, 2024 (CRD42024579161).

### Search Strategy

To identify relevant publications, we conducted systematic searches in the bibliographic databases Ovid Medline, Embase.com, and Web of Science (Core Collection) in collaboration with a medical information specialist. For OAGB—including studies using the terms mini gastric bypass or omega loop gastric bypass—databases were searched from inception to November 5, 2025. For RYGB and SG, searches were limited to January 1, 2020, to November 5, 2025, reflecting the update of the 2021 systematic review.

The search strategy used a combination of index terms and free-text words related to “weight loss,” “bariatric surgery,” “one anastomosis,” “mini gastric bypass,” and “omega loop.” Duplicate articles were excluded using EndNote X21.0.1 (Clarivate^tm^), following the Amsterdam Efficient Deduplication (AED)-method (Otten et al., 2019) and DedupEndNote (Lobbestael, 2023) [10, 11].

The full search strategies for all databases can be found in Supplementary material.

### Selection Process

Two reviewers (FH and KT) independently screened all potentially relevant titles and abstracts for eligibility using Rayyan [12]. Full-text articles were reviewed for the eligibility criteria when they could not be determined from the title and abstract. Disagreements were resolved through a consensus procedure with a third reviewer (RF).

Studies were included if they reported %TWL (or an equivalent metric clearly convertible to %TWL) in the text, tables, or graph legends, after LRYGB, SG, or OAGB, with a minimum cohort of 100 patients and a minimum follow-up of one year. All included studies reported outcomes in adults aged 18–65 years who met IFSO criteria for bariatric surgery, with baseline BMI ≥ 40 kg/m2, or ≥ 35 kg/m2 with obesity-related comorbidities.

Studies were excluded if they primarily involved open or revisional procedures. We also excluded studies that enrolled patients with a baseline BMI < 35 kg/m² or age < 18 or > 65 years. Data extracted solely from graphs were disregarded, unless explicitly clarified in the text or figure legend. Studies limited to very specific patient subgroups (e.g., defined by age, sex, or a single comorbidity such as type 2 diabetes) were excluded, because those factors could confound postoperative weight loss. If multiple reports clearly described overlapping patient cohorts (i.e., operated in the same center and time period), only the largest and most relevant dataset was included. Registry-based studies reporting aggregated outcomes were analyzed separately because they can reflect selection bias and may overlap with single-center cohorts. Reviews, letters, case reports, and comments were excluded.

### Data Assessment

The full text of the selected articles was obtained for further review. Two reviewers (FH and RA) independently evaluated the methodological quality of the full-text papers using the Newcastle–Ottawa Scale (NOS) quality assessment tool for observational studies. A modified scale was used for single-cohort studies [13].

### Statistical Analysis

Pooled %TWL values were calculated using sample-size–weighted means. The pooled standard deviation (SD) was derived from a pooled variance calculation incorporating within-study variance and between-study differences in study means. When %TWL was reported with 95% CI instead of SD, the SD was approximated assuming a normal distribution using the CI width and corresponding sample size. Studies without a reported measure of dispersion were included in pooled mean calculations but excluded from pooled SD estimates. No formal meta-analysis or comparative effect modeling was performed because of substantial clinical and methodological heterogeneity between studies. The pooled values and trendlines should therefore be interpreted as descriptive summaries of reported outcomes rather than comparative estimates of procedural effectiveness. We produced trendlines only to visually summarize overall patterns in reported %TWL over time. They should not be interpreted as predictive models.

## Results

**Fig. 1:**
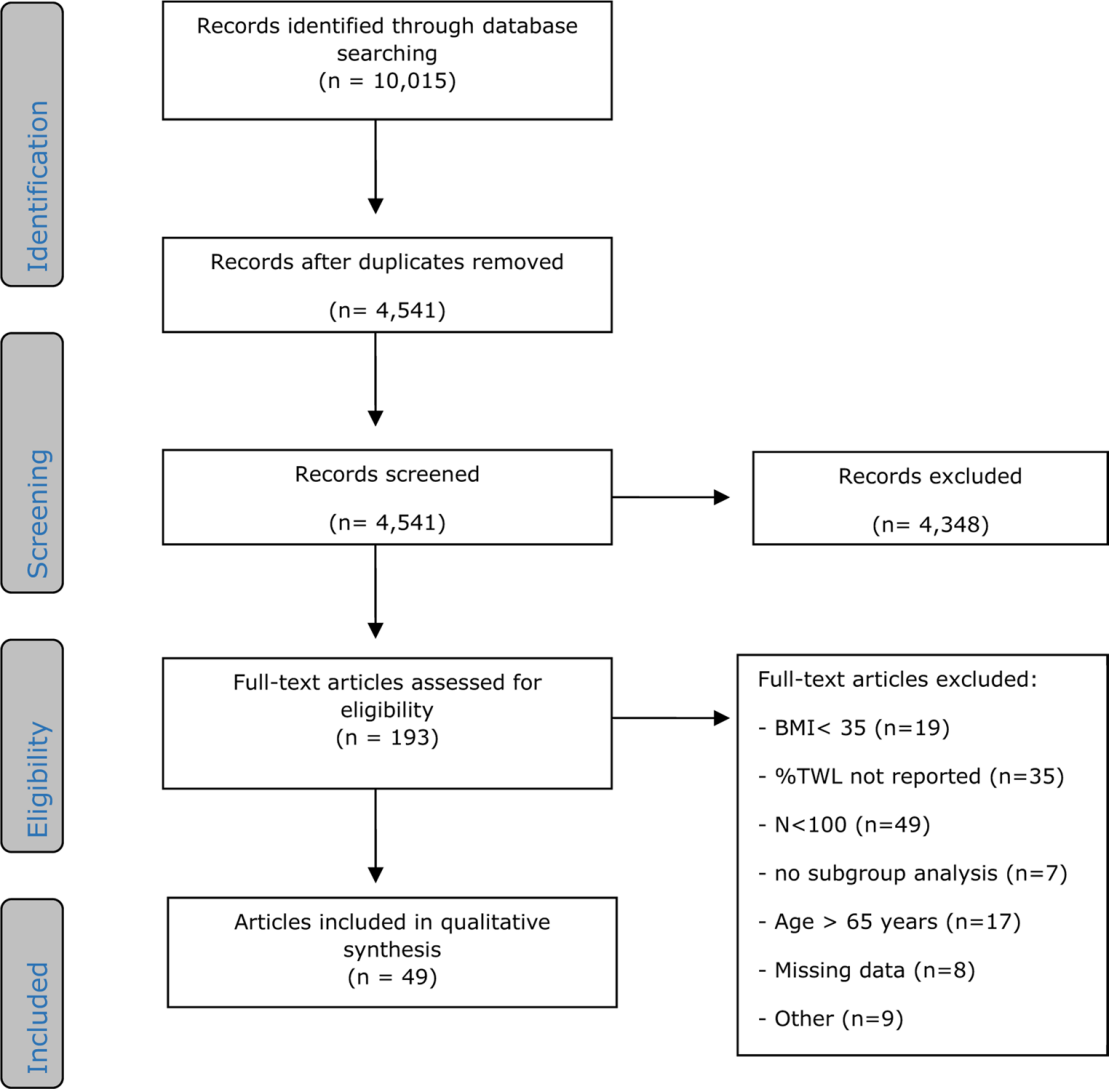
Flowchart of the search and selection procedure of studies

**Fig. 2:**
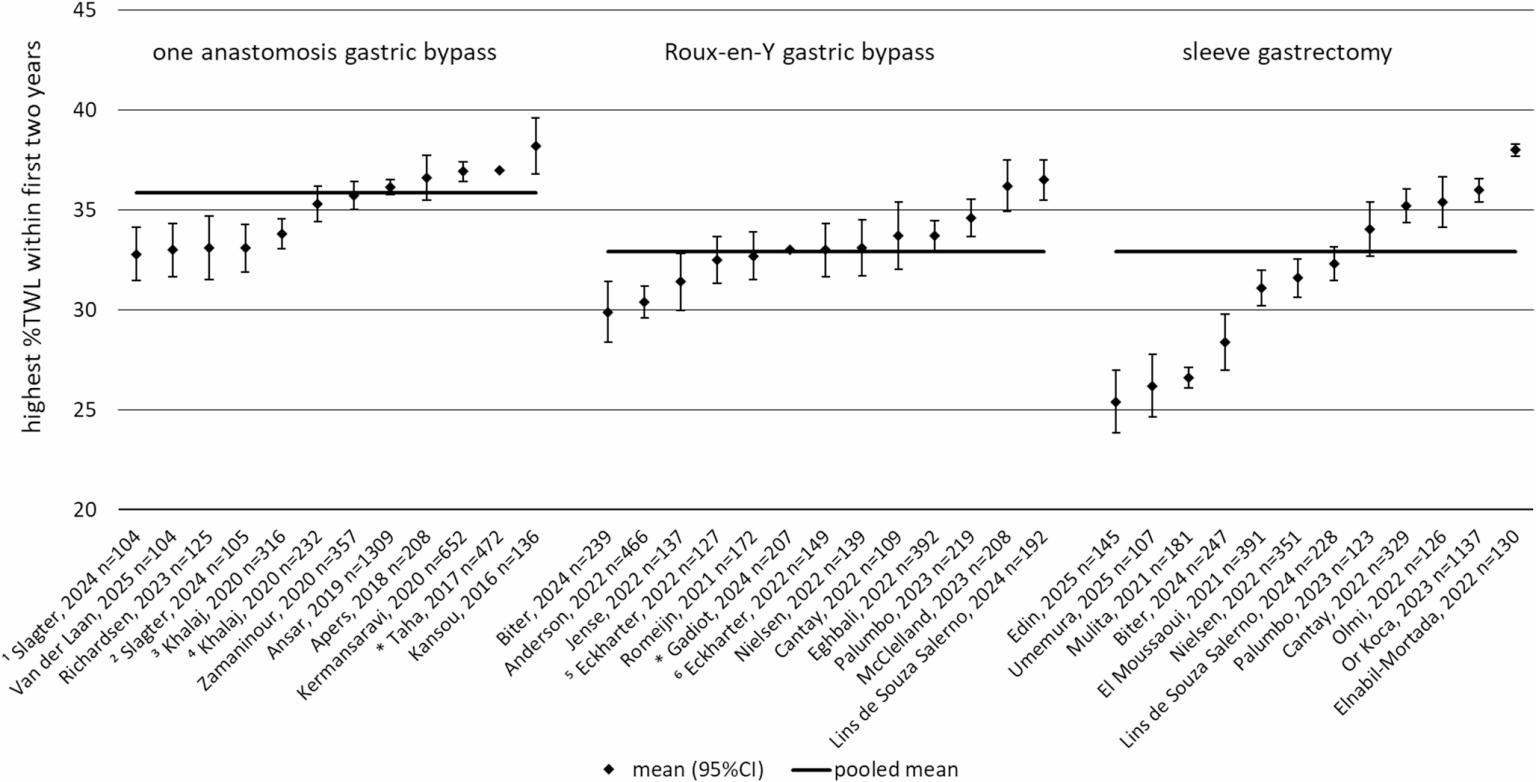
Mean nadir percentage total weight loss (%TWL) with 95% confidence intervals (CI) within the first two years after one anastomosis gastric bypass, Roux-en-Y gastric bypass and sleeve gastrectomy. Pooled means (full lines) 35.9%TWL, 32.9%TWL and 32.9%TWL respectively. * No information on CI available. 1) Subgroup biliopancreatic limb (BP) length 150cm. 2) Subgroup BP limb length 150-210cm. 3) Subgroup BP limb length 150cm. 4) Subgroup BP limb length 200cm. 5) Subgroup BP limb length 100cm. 6) Subgroup BP limb length 50cm

**Fig. 3:**
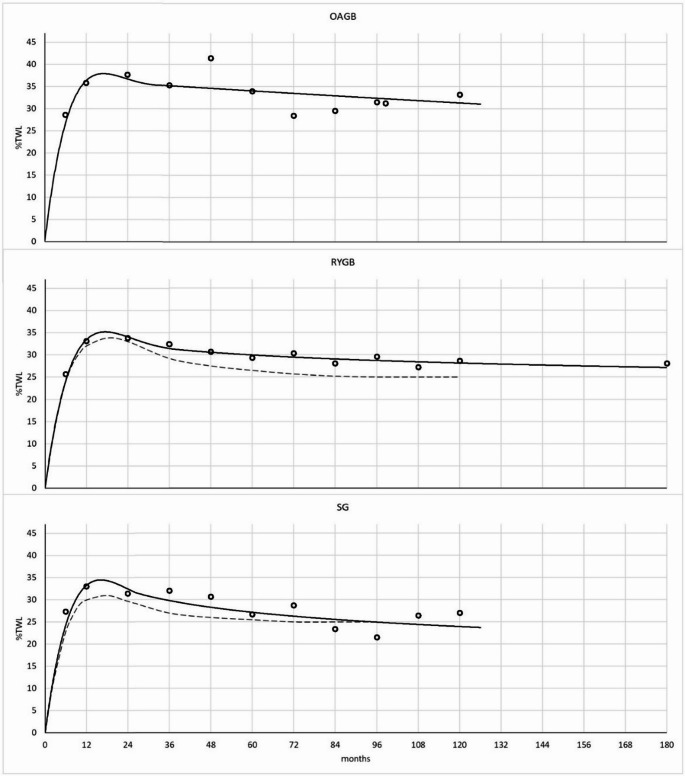
Pooled mean percentage total weight loss (%TWL) after one anastomosis gastric bypass (OAGB, *n* = 9922), Roux-en-Y gastric bypass (RYGB, *n* = 13078) and sleeve gastrectomy (SG, *n* = 9243) from 75 studies published until 2025. Full lines: descriptive polynomial (2–3 years) and logarithmic (> 3 years) trendlines. Dotted lines: results from previous systematic review by van Rijswijk et al., 2021

The literature search generated a total of 10,015 references: 3,125 in Ovid Medline, 3,765 in Embase.com, and 3,125 in Web of Science. After removing duplicates, 4,541 references remained. A total of 49 studies were included for analysis. The flowchart of the search and selection process is presented in Fig. 1. Nine studies reported registry-based data, comprising 119,277 patients after RYGB, 34,635 after SG, and 860 after OAGB. Of the remaining studies, 21 reported on RYGB, 18 on SG, and 16 on OAGB. Three studies reported on both OAGB and RYGB, one on OAGB and SG, seven on RYGB and SG, and one on all three procedures. Seven randomized controlled trials were identified. Table 1 shows baseline characteristics. Table 2 presents %TWL outcomes. Figure 2 summarizes the highest reported %TWL within 24 months (used as a proxy for nadir) of all studies that reported %TWL at one and/or two years after OAGB, RYGB, and SG. Pooled means are indicated by solid lines showing 35.9%TWL, 32.9%TWL, and 32.9%TWL, respectively. Figure 3 shows descriptive pooled mean %TWL during all available (non-registry data) follow-up after OAGB (*n* = 6,782), RYGB (*n* = 6,925), SG (*n* = 5,217). These trendlines are intended to illustrate overall patterns in reported %TWL rather than comparative or predictive models. Quality assessment of all included studies using the NOS is presented in Table 3, with NOS scores ranging from six to nine points, representing fair-to-good quality.

**Table 1:** Baseline data of studies included in the systematic review

Author, year of publication	Country	Study design	Study size	Number included	BP limb length	% male	Mean BMI (SD)	Mean age	T2DM (%)
OAGB									
Abu-Abeid, 2024 [14]	Israel	Retrospective	424	363	180–200 cm	41.6	42 (5.2)	42	26.0
Al-Hasani, 2025 [15]	UK	Retrospective	101	101	150 cm	9.9	47.2 (6.6)	44	22.7
Ansar, 2019 [16]	Iran	Prospective	1701	1309	100–280 cm	22.6	49.6 (6.4)	39	15.2
Apers, 2018 [17]	Netherlands	Prospective	287	287	200 cm	14.6	42.7 (5.8)	44	23.0
Hatami, 2023 [18]	Iran	Retrospective	2567	1381	NR	21.7	46.6 (6.5)	39	23.5
Kansou, 2016 [19]	France	Retrospective	272	136	200 cm	6.6	42.8 (5.0)	41	19.9
Kermansaravi, 2020 [20]	Iran	Retrospective	653	653	150–220 cm	18.4	44.4 (M)	37 (M)	17.0
Khalaj, 2020 [21]	Iran	Retrospective	800	383	160 cm	14.9	45.6 (6.3)	40	34.7
Khalaj, 2020 [22]	Iran	Retrospective	800	272	200 cm	15.1	46.7 (6.4)	39	40.4
Liagre, 2022 [22]	Italy	Retrospective	940	335	150 cm	20.3	42.8 (5.3)	38	NR
Neuberg, 2019 [23]	France	Retrospective	295	163	150 cm	20.9	40.3 (6.0)	41	13.0
Richardsen, 2023 [24]	Norway	Retrospective	147	125	170–200 cm	35.2	134.4 kg*	47	38.3
Robert, 2024 [25]	France	RCT	253	114	200 cm	25.4	44.0 (6.1)	44	26.5
Slagter, 2024 [26]	Netherlands	RCT	212	105	150 cm	10.5	41.1 (3.7)	43	14.3
Slagter, 2024 [26]	Netherlands	RCT	212	107	150–210 cm	13.1	40.5 (3.6)	45	13.1
Taha, 2017 [27]	Egypt	Retrospective	472	472	150–300 cm	26.1	46.8 (7.2)	42	98.5**
van der Laan, 2025 [28]	Netherlands	Retrospective	485	372	150–200 cm	18.6	42 (4.0)	46	21.2
Zamaninour, 2020 [29]	Iran	Retrospective	363	363	140–280 cm	17.9	46.7 (6.3)	40	22.9
RYGB									
Anderson, 2022 [30]	USA	Retrospective	768	581	NR	18	47.3 (8.1)	45	35.0
Barberá-Carbonell 2025 [31]	Switzerland	Prospective	944	944	50–150 cm	24.0	45.7 (5.9)	39	20.1
Biter, 2024 [32]	Multi (Netherlands)	RCT	628	316	NR	17.7	43.3 (4.7)	43	62.0
Cantay, 2022 [33]	Turkey	Retrospective	534	109	NR	32.4	44.8 (5.6)	42	30.9
Eghbali, 2022 [34]	Iran	Retrospective	410	410	NR	20	45.8 (5.5)	40	15.0
Eckharter, 2022 [35]	Switzerland	Retrospective	342	132	100 cm	21	40.9 (3.9)	42	18.0
Eckharter, 2022 [35]	Switzerland	Retrospective	342	176	50 cm	18	42.4 (4.3)	42	14.0
Gadiot, 2024 [36]	Netherlands	RCT	444	222	60 cm	16.7	42.3 (4.4)	42	23.4
Hatami, 2023 [18]	Iran	Retrospective	2567	870	NR	16.4	45.1 (5.2)	40	15.5
Jense, 2022 [37]	Netherlands	Retrospective	184	184	NR	20.1	44.7 (5.5)	43	24.6
Kraljević, 2025 [38]	Switzerland	RCT	217	110	50 cm	28.2	44.2 (5.3)	42	26.1***
Liagre, 2022 [22]	Italy	Retrospective	940	432	NR	17.4	42.7 (4.7)	40	NR
Lins de Souza Salerno, 2024 [39]	Brazil	Retrospective	591	327	NR	15.0	42.2 (M)	40	19.6
Luo, 2022 [40]	China	Retrospective	380	380	NR	19.7	47.3 (0.4)	44	39.2
McClelland, 2023 [41]	USA	Prospective	486	486	NR	11.3	48.4 (6.5)	36 (M)	24.3
Nielsen, 2022 [42]	Norway	Prospective	580	167	NR	25.1	43.1 (5.0)	42	8.4
Palumbo, 2023 [43]	Italy	Retrospective	792	452	NR	25.4	46.3 (5.8)	47	NR
Robert, 2024 [25]	France	Prospective	253	118	50 cm	21.2	44.0 (5.1)	42	26.7
Romeijn, 2021 [44, 45]	Netherlands	Retrospective	302	172	NR	16.3	42.4 (5.0)	45	NR
Salminen, 2022 [45]	Finland	RCT	240	119	NR	32.8	46.4 (5.9)	48	41.2
van der Laan, 2025 [28]	Netherlands	Retrospective	485	113	80–180 cm	14.2	42.0 (4.0)	46	24.8
Zerrweck, 2021 [46]	Mexico	RCT	210	105	50 cm	12.4	43.6 (6.0)	39	40.0
SG									
Ben-Porat, 2021 [47]	Israel	Retrospective	212	212	N/A	30.7	42.2 (4.9)	40	18.9
Biter, 2024 [32]	Multi (Netherlands)	RCT	628	312	N/A	18.6	43.7 (4.8)	43	54.0
Cantay, 2022 [33]	Turkey	Retrospective	534	329	N/A	45.9	46.0 (5.6)	41	37.4
Deregowska, 2021 [48]	Poland	Retrospective	218	174	N/A	25.29	44.0 (6.0)	42	NR
Edin, 2025 [49]	France	Retrospective	181	145	N/A	23.5	44.1 (NR)	44	31.7
El Moussaoui, 2021 [50]	Belgium-France	Prospective	529	529	N/A	24.6	42.9 (5.5)	41	15.7
Elnabil-Mortada, 2022 [51]	Egypt	Prospective	170	170	N/A	26.5	47.9 (8.2)	36	40.0
Hatami, 2023 [18]	Iran	Retrospective	2567	316	N/A	21.2	45.4 (6.5)	38	6.6
Kraljević, 2025 [38]	Switzerland	RCT	217	107	N/A	28.0	43.6 (5.3)	43	19.2***
Lins de Souza Salerno, 2024 [39]	Brazil	Retrospective	591	264	N/A	19.3	41.1 (M)	40	6.8
Luo, 2022 [40]	China	Retrospective	334	334	N/A	22.5	49.9 (0.5)	45	32.3
Mulita, 2021 [52]	Greece	Retrospective	209	209	N/A	25.8	43.2 (2.8)	34	NR
Nielsen, 2022 [42]	Norway	Prospective	580	376	N/A	25	42.8 (5.3)	43	13.8
Olmi, 2022 [53]	Italy	RCT	278	140	N/A	28.4	45.2 (7.0)	41	8.6
Or Koca, 2023 [54]	Turkey	Retrospective	1332	1137	N/A	17.1	45.9 (M)	39 (M)	13.3
Palumbo, 2023 [43]	Italy	Retrospective	792	235	N/A	36.2	51.6 (8.5)	48	NR
Salminen, 2022 [45]	Finland	RCT	240	121	N/A	28.1	45.5 (6.2)	49	43.0
Umemura, 2025 [55]	Japan	Retrospective	107	107	N/A	44.9	43.4 (6.1)	43	49.5
Registries									
OAGB									
van der Laan, 2024 [56]	Netherlands	DATO****	12,929	860	150–180 cm	24.8	43 (M)	47 (M)	23.8
RYGB		Registry							
Arterburn, 2021 [57]	USA	Washington & California	31,158	17,258	NR	17.9	45.1 (7.2)	46	39.7**
Axer, 2023 [58]	Sweden	SOReg	60,426	52,570	NR	24.3	42.6 (5.4)	41	35.0
Brismann, 2021 [59]	Sweden	SOReg	10,633	5936	NR	20.9	42.9 (5.1)	40	15.1
Sundbom, 2025 [60]	Sweden	Prospective	29,578	29,578	NR	24.4	42.7 (5.6)	41	14.5
van der Laan, 2024 [28]	Netherlands	DATO****	12,929	860	60–80 cm	23.8	43 (M)	46 (M)	20.1
Winckelmann, 2022 [61]	Denmark	DBSO	16,053	13,075	NR	25.2	44.2 (M)	41 (M)	21.6
SG									
Al-Tai, 2024 [62]	Sweden	SOReg	12,117	9360	N/A	21.3	40.4 (3.7)	41	9.5
Arterburn, 2021 [57]	USA	Washington & California	31,158	13,900	N/A	19.1	43.6 (6.5)	45	26.1**
Axer, 2023 [58]	Sweden	SOReg	60,426	7856	N/A	19.6	39.6 (5.6)	41	21.1
Gormsen, 2024 [63]	Denmark	DOSR	5438	541	N/A	29.6	44.6 (M)	42 (M)	24.4
Winckelmann, 2022 [61]	Denmark	DBSO	16,053	2978	N/A	22.1	43.1 (M)	42 (M)	13.3

*BP* biliopancreatic, *DATO* dutch audit for treatment of obesity, *DBSO* Danish quality registry for treatment of severe obesity, *DOSR*danish obesity surgery registry; *M*median*,N/A*not applicable,* NR* not reported, *RCT* randomized controlled trial, *SD* standard deviation, *SOReg* Scandinavian obesity surgery registry, *T2DM* type 2 diabetes mellitus

*Only baseline kilograms were registered

**No differentiation in type I or II diabetes was made in this article

***Percentage T2DM at baseline only included patients who completed 10-year follow-up

****Prospective study design, all other registry studies had a retrospective study design

**Table 2:** Percentage total weight loss (%TWL) after one anastomosis gastric bypass (OAGB), Roux-en-Y gastric bypass (RYGB), and sleeve gastrectomy

Author, year of publication	BP limb length	6 months		1 year		2 years		5 years		10 years	
		*n*	Mean (SD)	*n*	Mean (SD)	*n*	Mean (SD)	*n*	Mean (SD)	*n*	Mean (SD)
OAGB											
Abu-Abeid, 2024	180–200 cm									363	31.3 (14.0)*
Al-Hasani, 2025	150 cm	91	28.2 (8.7)	88	35.9 (9.1)	82	37.3 (9.3)				
Ansar, 2019	100–280 cm			1309	36.1 (7.0)						
Apers, 2018	200 cm			277	35.4 (7.3)	208	36.6 (8.3)				
Hatami, 2023	NR							1077	34.6 (8.4)		
Kansou, 2016	200 cm			136	38.2 (8.4)						
Kermansaravi, 2020	150–220 cm	622	29.3 (M)	652	36.9 (M)						
Khalaj, 2020	160 cm			316	33.8 (6.7)						
Khalaj, 2020	200 cm			232	35.3 (6.9)						
Liagre, 2022	150 cm									335	33.2 (10.0)
Neuberg, 2019	150 cm							163	27.7 (4.7)**		
Richardsen, 2023	170–200 cm					125	33.1 (9.1)				
Robert, 2024	200 cm							87	31.3 (11.2)		
Slagter, 2024	150 cm			104	32.8 (6.9)						
Slagter, 2024	150–210 cm			105	33.1 (6.2)						
Taha, 2017	150–300 cm	472	27.7 (NR)**	472	37.0 (NR)**						
van der Laan, 2025	150–200 cm			104	33 (7)			81	30 (10)		
Zamaninour, 2020	140–280 cm			357	35.7 (6.9)						
RYGB											
Anderson, 2022	NR			466	30.4 (8.6)			114	28.1 (13.1)		
Barberá-Carbonell, 2025	50–150 cm									866	28.6 (NR)
Biter, 2024	NR			239	29.9 (NR)			239	26.0 (NR)		
Cantay, 2022	NR			109	33.7 (9.0)						
Eghbali, 2022	NR			400	32.1 (7.7)	392	33.69 (7.7)	318	28.8 (9.0)		
Eckharter, 2022	100 cm	127	26.3 (5.6)	127	32.5 (7.4)	119	32.4 (8.4)				
Eckharter, 2022	50 cm	164	25.4 (5.1)	158	32.2 (6.8)	149	33.0 (8.3)				
Gadiot, 2024	60 cm			207	33.0 (NR)	173	32.6 (NR)	136	28.6 (NR)		
Hatami, 2023	NR							704	29.1 (10.0)		
Jense, 2022	NR			150	30.4 (7.0)	137	31.4 (8.6)	135	27.6 (9.0)		
Kraljević, 2025	50 cm									73	27.5 (10.6)
Liagre, 2022	NR									320	29.5 (10.9)
Lins de Souza Salerno, 2024	NR			322	35.5 (CI 34.8; 36.1)	192	36.5 (CI 35.5–37.5)	74	31.8 (CI 29.9–33.7)		
Luo, 2022	NR							380	31.2 (0.4)		
McClelland, 2023	NR	326	27 (5.3)	366	35.4 (7.9)	208	36.2 (9.5)	181	33.4 (9.8)	171	28.6 (11.4)
Nielsen, 2022	NR			154	32.8 (NR)	139	33.1 (NR)	119	27.9 (NR)		
Palumbo, 2023	NR	432	25.0 (NR)	376	32.6 (NR)	219	34.6 (NR)	51	31.6 (NR)		
Robert, 2024	50 cm							84	29.2 (10.8)		
Romeijn, 2021	NR					172	32.7 (8.0)				
Salminen, 2022	NR									95	26.9 (NR)
van der Laan, 2025	80–180 cm			99	29 (9)			88	27 (11)		
Zerrweck, 2021	50 cm			96	33.5 (6.4)						
SG											
Ben-Porat, 2021	N/A									212	21.7(10.7)****
Biter, 2024	N/A			247	28.4 (NR)			247	22.5 (NR)		
Cantay, 2022	N/A			329	35.2 (7.7)						
Deregowska, 2021	N/A			129	31.2 (10.0)	49	35.5 (9.8)				
Edin, 2025	N/A					145	25.4 (9.6)				
El Moussaoui, 2021	N/A			391	31.1 (8.8)**			285	33.2 (5.6)		
Elnabil-Mortada, 2022	N/A			155	37.1 (5.7)	130	38.0 (1.7)				
Hatami, 2023	N/A							268	26.2 (12.4)		
Kraljević, 2025	N/A									69	26.3 (13.6)
Lins de Souza Salerno, 2024	N/A			228	32.3 (CI 31.4–33.1)	135	30.7 (29.2–32.2)	43	23.4 (CI 20.2–26.6)		
Luo, 2022	N/A							334	25.1 (0.5)		
Mulita, 2021	N/A			197	26.3 (3.5)**	181	26.6 (3.6)*	103	28.2 (4.5)**		
Nielsen, 2022	N/A			351	31.6 (NR)	221	29.4 (NR)	257	25.2 (NR)		
Olmi, 2022	N/A			126	35.4 (7.2)						
Or Koca, 2023	N/A			1137	36.0 (NR)						
Palumbo, 2023	N/A	225	25.1 (NR)	190	32.4 (NR)	123	34.1 (NR)	22	29.75 (NR)		
Salminen, 2022	N/A									98	23.4 (NR)*
Umemura, 2025	N/A			107	26.2 (8.2)						
Registries											
OAGB											
van der Laan, 2024	150–180 cm			757	34.0 (7.0)	570	35.1 (8.1)	349	30.0 (9.7)		
RYGB											
Arterburn, 2021	NR			14,487	28.4 (NR)			6251	21.7 (NR)	174	20.2 (NR)
Axer, 2023	NR					52,570	32.3 (8.8)				
Brismann, 2021	NR							5936	29.1 (9.8)		
Sundbom, 2025	NR			26,285	31.5 (7.7)	20,074	31.9 (9.0)	16,787	28.0 (10.0)	9966	24.6 (11.0)
van der Laan, 2024	60–80 cm			709	32.7 (6.7)	532	33.4 (8.2)	300	28.8 (9.0)		
Winckelmann, 2022	NR			11,265	33 (M)	4763	34.4 (M)				
SG											
Al-Tai, 2024	N/A			7136	27.6 ± 8.3						
Arterburn, 2021	N/A			9840	23.0 (NR)			899	16.0 (NR)		
Axer, 2023	N/A					7856	25.6 (9.8)				
Gormsen, 2024	N/A			494	28.4 (M)	413	27.3 (M)				
Winckelmann, 2022	N/A			1809	28.6 (M)	600	27.4 (M)				

*CI* 95% confidence interval,* BP* biliopancreatic, *FU* follow-up, *M* median, *N/A* not applicable, *NR* not reported, *SD* standard deviation

*Follow-up was 98.5 months

**Only BMI change was reported during FU

***Follow-up was 36 and 108 months

****Follow-up was 96 months

**Table 3:** Quality assessment of studies, using the Newcastle-Ottawa Scale

	Selection criteria	Comparability	Outcomes	Total
OAGB				
Abu-Abeid, 2024	****	*	**	*******
Al-Hasani, 2025	***	*	***	*******
Ansar, 2019	***	**	***	********
Apers, 2018	***	*	***	*******
Kansou, 2016	****	**	***	*********
Kermansaravi, 2020	****	**	***	*********
Khalaj, 2020	****	*	***	********
Neuberg, 2019	***		**	*****
Richardsen, 2023	***		***	******
Slagter, 2024	****	**	***	*********
Taha, 2017	***		***	******
Zamaninour, 2020	***		***	******
RYGB				
Anderson, 2022	****	**	***	*********
Barberá-Carbonell, 2025	****	**	***	*********
Eckharter, 2022	****	**	***	*********
Eghbali, 2022	***	**	***	********
Gadiot, 2024	****	**	***	*********
Jense, 2022	****		***	*******
McClelland, 2023	***	*	***	*******
Romeijn, 2021	***	**	**	*******
Zerrweck, 2021	****	**	***	*********
SG				
Ben-Porat, 2021	***	*	***	*******
Deregowska, 2021	***	*	**	******
Edin, 2025	****		***	*******
El Moussaoui, 2021	***	**	**	*******
Elnabil-Mortada, 2022	****	**	***	*********
Mulita, 2021	***		***	******
Olmi, 2022	****	**	***	*********
Or Koca, 2023	***	**	***	********
Umemura, 2025	****	**	***	*********
OAGB and RYGB				
Liagre, 2022	****	**	***	*********
Robert, 2024	****	**	***	*********
van der Laan, 2024	****	**	***	*********
RYGB and SG				
Biter, 2024	****	**	***	*********
Kraljevic, 2025	****	**	***	*********
Lins de Souza Salerno, 2024	****	*	**	*******
Luo, 2022	****	**	***	*********
Nielsen, 2022	****	**	***	*********
Palumbo, 2023	****	*	**	*******
Salminen, 2022	****	**	***	*********
OAGB, RYGB and SG				
Hatami 2023	****	**	***	*********
Registry studies				
Al-Tai, 2024	****	**	***	*********
Arterburn, 2021	****	**	***	*********
Axer, 2023	****	**	***	*********
Brismann, 2021	***	**	***	********
Gormsen, 2024	***		***	******
van der Laan, 2024	****	**	***	*********
Winckelmann, 2022	****		**	******
Sundbom, 2025	***	**	***	********

### Outcomes after One Anastomosis Gastric Bypass

The percentage of males ranged from 6.6% to 41.6%, mean age from 37 to 47 years, and mean BMI from 40.3 kg/m2 to 49.6 kg/m2. Biliopancreatic limb (BP) length varied between 100 and 300 centimeters. In nine studies, limb length was variable, in six studies a fixed BP limb length was reported, and in one study BP length was not reported. In the study of Khalaj et al. (2020) and Slagter et al.(2024), a distinction between two subgroups was made based on the technique that was used. Consistent with the data presented in those publications, outcomes were reported separately for the subgroups in this review. Weight loss results are summarized in Table 2. The nadir %TWL, defined as the highest weight loss within two years from surgery, was reported by 11 out of 18 studies, with a pooled mean %TWL of 35.9 ± 8.2%. One study didn’t report a standard deviation (SD) and was excluded from the pooled analysis. Three studies (*n* = 1,327) reported %TWL after five years, with a pooled mean %TWL of 33.4 ± 8.4%. The longest follow-up was provided by Liagre et al., 10 years after primary surgery (*n* = 335), with a %TWL of 33.2 ± 10%.

### Outcomes after Roux-en-Y Gastric Bypass

The proportion of male patients ranged from 11.3% to 32.8%, mean age from 36 to 48 years, and mean baseline BMI from 40.9 to 48.4 kg/m². Biliopancreatic limb length was not reported in a substantial number of studies. Where specified, BP limb length ranged from 50 to 180 cm. In the study of Eckharter (2022), outcomes were reported separately for subgroups with different limb lengths. In line with the original publications, these subgroups were treated as separate cohorts in this review. The nadir %TWL was reported by 17 out of 21 studies, with a pooled mean %TWL of 32.9 ± 8.6%. Thirteen studies reported 5-year follow-up %TWL, including a total of 2,487 patients. The pooled mean %TWL and SD at 5 years was 29.3 ± 9.7%. One study did not report a SD and was therefore excluded from the pooled SD calculation. Four studies reported 10-year follow-up %TWL, comprising a total of 659 patients. The pooled mean %TWL and SD at 10 years was 28.6 ± 11%. One study reported 15-year follow-up %TWL, including 92 patients, with a mean %TWL of 28.0 ± 13.0%.

### Outcomes after Sleeve Gastrectomy

The proportion of male patients ranged from 17.1% to 44.9%, mean age from 34 to 49 years, and mean baseline BMI from 41.1 to 51.6 kg/m². Highest %TWL within the first 24 months was reported by 13 studies, with a pooled mean of 32.9 ± 8.6%. Five-year follow-up %TWL was reported by eight studies, including a total of 1,559 patients, with a pooled mean %TWL of 26.6 ± 9.6%TWL. Two studies reported %TWL at the 10-year follow-up interval, comprising 167 patients. One study reported median (IQR) values; the median was assumed to approximate the mean and the SD was estimated from the IQR. The pooled mean %TWL was 24.6 ± 10.1%.

### Outcomes of Registry Studies

One registry-based study reporting on OAGB was included, comprising 860 patients. The proportion of male patients was 24.8%, with a median age of 47 years and a median baseline BMI of 43 kg/m². For RYGB, six registry studies were included, comprising a total of 119,277 patients. The proportion of male patients ranged from 17.9% to 25.2%, mean or median age from 40 to 46 years, and mean baseline BMI from 42.6 to 45.1 kg/m². For SG, five registry studies were included, comprising 34,635 patients. The proportion of male patients ranged from 19.1% to 29.6%, mean or median age from 41 to 45 years, and mean baseline BMI from 39.6 to 44.6 kg/m².

For OAGB, the highest %TWL within the first 24 months was reported in a single study, with a mean %TWL of 35.1 ± 8.1% at 24 months. For RYGB, pooling the highest reported %TWL per cohort within the first 24 months yielded a pooled mean highest %TWL of 32.6 ± 8.6%. For SG, the pooled mean highest %TWL within the first 24 months was 25.9 ± 8.4%.

The longest follow-up was 5 years for OAGB, resulting in a %TWL of 30.0 ± 9.7% in a cohort of 349 patients. For RYGB, the longest follow-up was 10 years (2 studies), with a pooled mean %TWL of 24.5 ± 10.9%. One study reported 5-year follow-up for patients after SG, showing %TWL of 16.0 ± 9.0%.

## Discussion

This updated systematic review shows that descriptively pooled early %TWL is highest after OAGB, whereas early %TWL is similar after RYGB and SG. At longer-term follow-up, %TWL also appears more favorable after OAGB, although long-term evidence remains limited. To our knowledge, this is the first systematic review to comprehensively summarize %TWL after OAGB. These findings are consistent with previous studies suggesting greater or comparable weight loss after OAGB relative to RYGB [64–70]. Previous studies also compared OAGB to SG, but in those studies results were expressed as excess weight loss [71, 72]. For RYGB and SG, we showed that studies published in the past 5 years yield similar weight loss charts compared to previous studies, as trend lines for %TWL during follow-up are in line with the systematic review of Van Rijswijk et al. (Fig. 3). These findings should not be interpreted as indicating overall superiority of OAGB. The pooled values presented in this review are descriptive weighted averages derived from heterogeneous observational cohorts with substantial variation in patient selection, operative technique, limb lengths, follow-up completeness, and outcome reporting. They cannot be interpreted as a meta-analysis comparing the effectiveness between procedures. Furthermore, weight loss represents only one dimension of postoperative outcome. Comprehensive evaluation of bariatric procedures should additionally include remission of obesity-related comorbidities, nutritional consequences, gastrointestinal symptoms, revisional surgery, complications, and long-term safety.

A major limitation is the limited availability of long-term data, particularly for OAGB and SG. While RYGB outcomes are supported by more extensive follow-up, long-term evidence for OAGB and SG remains sparse. Attrition during long-term follow-up may have resulted in overestimation of durable weight loss outcomes, particularly in studies with limited follow-up completeness. Furthermore, weight loss alone does not capture the full long-term impact of a specific procedure, as complications such as reflux, dumping, ulceration, hypoglycemia, obstruction, nutritional deficiencies, and revisional surgery can substantially influence long-term patient outcomes. Revisional procedures may influence long-term weight trajectories, but revisional cohorts were excluded from this review to preserve comparability with primary procedures. Comparisons between OAGB, RYGB, and SG may also have been influenced by differing search timeframes, as earlier SG and RYGB studies included in the previous review were not part of the present update. Substantial heterogeneity in surgical technique and reporting further limits interpretation. Biliopancreatic limb length varied widely in OAGB studies and was inconsistently reported for RYGB. For SG, technical variables such as bougie size and extent of resection were rarely standardized. These differences likely contribute to variability in reported %TWL and complicate direct comparison between procedures [20, 62, 73].

Overall, %TWL is a useful and increasingly standardized metric for reporting weight loss after MBS. However, improved standardization of surgical technique reporting and more high-quality studies with extended follow-up are needed to enhance comparability and interpretation of long-term outcomes.

## Conclusion

This updated systematic review provides a descriptive summary of %TWL outcomes after OAGB, RYGB, and SG. Descriptive pooled estimates suggest that early %TWL is highest after OAGB, while early %TWL is similar after RYGB and SG. Long-term %TWL also appears more favorable after OAGB, although long-term evidence remains limited, particularly for OAGB and SG. Standardized reporting of %TWL and surgical technique, as well as high-quality studies with extended follow-up, are needed to improve comparison of long-term outcomes across bariatric procedures.

## Data Availability

No datasets were generated or analysed during the current study.
